# Early intervention in systemic lupus erythematosus: time for action to improve outcomes and health-care utilization

**DOI:** 10.1093/rap/rkab106

**Published:** 2021-12-28

**Authors:** Md Yuzaiful Md Yusof, Edward M Vital

**Affiliations:** 1 Leeds Institute of Rheumatic and Musculoskeletal Medicine, University of Leeds; 2 NIHR Leeds Biomedical Research Centre, Leeds Teaching Hospitals NHS Trust, Leeds, UK

Although new therapies have been licensed for the treatment of SLE, we do not yet know to what extent these will improve mortality, quality of life and health-care costs. To date, these therapies have mostly been trialled and prescribed to patients with an SLE diagnosis of several years, and after other immunosuppressants. But is this the best time to use our most effective therapies? In this issue of *Rheumatology Advances in Practice*, Samnaliev *et al.* [1] conducted a retrospective cohort analysis of direct health-care utilization of adults with SLE in the UK using the Clinical Practice Research Datalink (CPRD)–Hospital Episode Statistics (HES) database for >10 years. Their data suggest that, unlike some other diseases, the early phases of SLE might represent the greatest unmet need to suppress inflammation and prevent irreversible damage.

Previous work has already suggested this. The standardized mortality ratio is highest in the first year of SLE diagnosis, at 5.4 [2]. This is attributed to the delay from symptom onset to diagnosis, which can be as long as 6.4 years [3]. Long-term complications, such as cardiovascular disease, are increased up to 50 times, particularly given that therapy with glucocorticoids worsens long-term outcomes [4]. Furthermore, SLE impairs quality of life significantly, comparable to other chronic diseases such as diabetes, hypertension and heart failure [5]. In order to acknowledge completely the overall disease burden, it is imperative to assess its economic implication, because there are limited data evaluating health-care utilization associated with SLE over time.

The CPRD used in the study by Samnaliev *et al.* [1] captures data from a large number of patients in real-world clinical practice. However, extraction of accurate information from CPRD can be challenging. Eight hundred and two patients were identified and analysed. Disease severity was defined from the routine clinical data based on treatment intensity (e.g. prednisolone ≥60 mg/day or CYC) and key co-morbid conditions, such as end-stage renal disease. These are insensitive indicators, therefore suggesting that they captured the most severe lupus [1].

Samnaliev *et al.* [1] reported several important findings. First, there was an incremental increase per year in the mean unadjusted direct health-care cost in the 3 years before diagnosis, which was attributed to the costs of primary care and prescriptions [1]. This concurred with a previous study, which also used the CPRD and reported that patients with SLE had a significantly higher consultation rate than healthy controls (median 9.2 *vs* 3.8 per year respectively) in the 5 years preceding diagnosis, which was attributable, in part, to clinical features of SLE [6]. In the paper by Samnaliev *et al.* [1], the rise in the mean (S.d.) unadjusted direct health-care cost peaked in the first year after diagnosis at £7532 (£9634) per patient and remained high for the next 9 years. In the first year after diagnosis alone, the adjusted total mean annual increase in costs per patient was £4476 (95% CI £3809, £5143) greater compared with the year before the diagnosis. Second, the authors used models of individual trajectories of mean all-cause health-care costs and reported that the increase in costs per year was 4.7- and 1.6-fold higher among patients with severe SLE compared with those who had mild or moderate disease [1]. Third, in another paper published by the same group and using the same methodology in this issue of *Rheumatology Advances in Practice*, Langham *et al.* [7] suggested that there was some delay in the initiation of SLE treatment following diagnosis, with the mean (S.d.) time to initiation of any medication being 177 (385.3) days. Consequently, almost all patients (750 of 802; 93.5%) experienced at least one flare episode during the follow-up. In year 1 of diagnosis, the mean (S.d.) overall flare rate was 3.5 (2.5), and the median time to first flare was 63 (95% CI 57, 71) days. Patients with moderate or severe disease had the shortest median time to first flare compared with those who had mild SLE [7].

Collectively, these findings present a concerning picture. Not only do highly severe manifestations present early in the disease and impact on quality of life and mortality, but also these diagnoses are made late. This suggests that a radically different approach to referral, diagnosis and intensity of early treatment is warranted to improve outcomes, including the prevention of irreversible damage; the availability of a new therapy might not be sufficient unless the entire early treatment pathway is revised. In other diseases, such as RA, the 2016 EULAR guidelines advocate for patients with suspected inflammatory arthritis to be referred to, and seen by, a rheumatologist, within 6 weeks of the onset of symptoms [8]. Specific guidelines have been developed in individual health-care systems [9]. Therefore, national and international lupus experts and societies should collaborate and establish similar guidelines for patients with a suspected autoimmune connective tissue disease.

However, we must consider some limitations in both studies. The use of electronic health records depended on whether a diagnosis was coded accurately in both the primary and secondary care. Hence, a misclassification of SLE is still possible and is more likely for the mild patients, which might exaggerate differences between severe and mild patient groups. Furthermore, the severity of SLE could have been underestimated for the severe category and overestimated for the mild to moderate categories. For example, a criterion for severe disease was oral prednisolone ≥60 mg/day, whereas the intention to treat of >20 mg/day in other established indices, such as the BILAG-2004, would be considered as severe. The study also lacks age- and gender-matched healthy controls for comparison. Lastly, the study also did not examine the impact of ancestry and ethnicity, which are factors that are known to impact on lupus severity and health-care utilization patterns.

Future treatment innovations in SLE should focus on early diagnosis and treatment. A broad range of research questions must be answered. Better diagnostics during the pre-clinical and early phase [10] are needed, accompanied by clear referral guidelines. Poor prognostic markers, either clinical or based on biomarkers [11] (or both) could help in patient selection for more intensive therapies. New clinical trial designs will be required when the objective is the prevention of severe disease rather than response. Finally, a full health economic analysis would confer whether such a strategy is cost-effective enough from the perspective of the funders/taxpayers.

## Acknowledgements

M.Y.M.Y. is a Senior Research Fellow and is funded/supported by the the Wellcome Trust Institutional Strategic Support Fund Fellowship (204825/Z/16/Z) and the National Institute for Health Research (NIHR) Doctoral Research Fellowship (DRF-2014-07-155). E.M.V. is an NIHR Clinician Scientist (CS-2013-13-032). This article/paper/report also presents independent research funded/supported by the NIHR Leeds Biomedical Research Centre. The views expressed are those of the author(s) and not necessarily those of the NIHR or the Department of Health and Social Care.

*Funding:* No specific funding was received from any bodies in the public, commercial or not-for-profit sectors to carry out the work described in this article.

*Disclosure statement:* M.Y.M.Y. has received consultancy fees from Aurinia Pharmaceuticals. E.M.V. has received honoraria, consultancy fees and research grant support from Aurinia, Roche, GSK, Lilly, Novartis and AstraZeneca.

## Data availability statement

All data underlying this article are available in the manuscript.
